# Chronic postsurgical pain after minimally invasive adrenalectomy: prevalence and impact on quality of life

**DOI:** 10.1186/s12871-022-01696-4

**Published:** 2022-05-19

**Authors:** Esmee V. van Helden, Allon van Uitert, Kim I. Albers, Monique A. H. Steegers, Henri J. L. M. Timmers, Frank C. H. d’Ancona, Selina E. I. van der Wal, Gert Jan Scheffer, Christiaan Keijzer, Michiel C. Warlé, Johan F. Langenhuijsen

**Affiliations:** 1 Department of Surgery, Radboud University Medical Center, Geert Grooteplein Zuid 10, 6525 GA Nijmegen, The Netherlands; 2 Department of Anesthesiology, Radboud University Medical Center, Geert Grooteplein Zuid 10, 6525 GA Nijmegen, The Netherlands; 3 Department of Urology, Radboud University Medical Center, Geert Grooteplein Zuid 10, 6525 GA Nijmegen, The Netherlands; 4 Department of Anesthesiology, Amsterdam University Medical Center, De Boelelaan 1118, 1081 HV Amsterdam, The Netherlands; 5 Department of Internal Medicine, Radboud University Medical Center, Geert Grooteplein Zuid 10, 6525 GA Nijmegen, The Netherlands

**Keywords:** Chronic postsurgical pain, Minimally invasive adrenalectomy, Quality of life, Hypoesthesia, Risk factors

## Abstract

**Background:**

Minimally invasive adrenalectomy is the standard of care for small adrenal tumours. Both the transperitoneal lateral approach and posterior retroperitoneal approach are widely used and have been proven to be safe and effective. However, the prevalence of chronic postsurgical pain has not been specifically investigated in previous studies. The primary goal of this study was to identify the prevalence of chronic postsurgical pain after minimally invasive adrenalectomy.

**Methods:**

A cross-sectional study was performed among all consecutive patients who had undergone minimally invasive adrenalectomy in a single university medical centre. The primary outcome was the prevalence of chronic postsurgical pain. Secondary outcomes were the prevalence of localized hypoesthesia, risk factors for the development of chronic postsurgical pain, and the Health-Related Quality of Life. Three questionnaires were used to measure the prevalence and severity of chronic postsurgical pain, hypoesthesia, and Health-Related Quality of Life. Logistic regression analysis was performed to determine risk factors for development of chronic postsurgical pain.

**Results:**

Six hundred two patients underwent minimally invasive adrenalectomy between January 2007 and September 2019, of whom 328 signed informed consent. The prevalence of chronic postsurgical pain was 14.9%. In the group of patients with chronic postsurgical pain, 33% reported hypoesthesia as well. Young age was a significant predictor for developing chronic postsurgical pain. The prevalence of localized hypoesthesia was 15.2%. In patients with chronic postsurgical pain, Health-Related Quality of Life was significantly lower, compared to patients without pain.

**Conclusions:**

The prevalence of chronic postsurgical pain following minimally invasive adrenalectomy is considerable. Furthermore, the presence of chronic postsurgical pain was correlated with a significant and clinically relevant lower Health-Related Quality of Life. These findings should be included in the preoperative counselling of the patient. In the absence of evidence for effective treatment in established chronic pain, prevention should be the key strategy and topic of future research.

**Supplementary Information:**

The online version contains supplementary material available at 10.1186/s12871-022-01696-4.

## Background

Since the introduction in 1992, minimally invasive adrenalectomy (MIA) has become the standard of care for the management of small (≤7 cm) benign adrenal tumours and, in selected cases, for the treatment of small (≤6 cm) malignant tumours [1]. The transperitoneal lateral approach (TLA) and the posterior retroperitoneal approach (PRA) have both been proven to be safe and effective when compared with open adrenalectomy, with low morbidity and complication rates, decreased blood loss, less postoperative pain, shorter hospital stay, and improved cosmetic effects [2, 3]. Nevertheless, patients regularly report chronic postsurgical pain (CPSP) [4]. CPSP is defined as chronic pain that develops or increases in intensity after a surgical procedure or tissue injury and persists beyond the healing process, i.e. at least 3 months after surgery [5]. Since MIA is frequently performed worldwide, it is important to report these functional outcomes, because CPSP can have significant impact on quality of life and health care demand [6]. Predisposing factors described in literature for development of CPSP are the presence of pre-existing pain, early postoperative pain, psychological factors, the surgical procedure itself and patient characteristics, such as age and sex [7, 8].

The primary outcome of this study was the prevalence of CPSP. Secondary outcomes were the prevalence of hypoesthesia, risk factors for CPSP and the impact of CPSP on Health-Related Quality of Life (HRQoL).

## Methods

### Study design and patient population

All adult patients who underwent MIA in our hospital between February 2007 and September 2019 were included in a cross-sectional study. Patients were approached by phone between October 2019 and January 2020. PRA was introduced in our hospital in 2011. Patients with (presumably) benign tumours or pheochromocytomas ≤7 cm, and a body mass index (BMI) < 35 kg/m^2^ were eligible for PRA. In all other patients TLA was indicated. Two surgeons performed the procedures, both with more than 15 years of laparoscopic experience. Living patients were approached by telephone, subsequently written information was sent after which informed consent was obtained. All patients were asked to complete three questionnaires at the time of study. The study was approved by the research ethics committee of the Radboud University Nijmegen Medical Centre (2019–5500).

### Measures

There were four objectives in this study; (1) assessment of CPSP; (2) assessment of localized hypoesthesia; (3) analysis of possible risk factors for CPSP; and (4) the influence of CPSP on HRQoL. The primary outcome of this study was the prevalence of CPSP. The presence of CPSP was scored when patients replied “yes” to the question if they currently had pain which could be related back to their adrenalectomy, i.e. wound pain, pain at site of the scar, flank pain, referred shoulder pain. When patients answered “yes” to the chronic pain question, they were included in the chronic pain group, and they were asked to fill in the follow up questionnaire: the Dutch version of the McGill Pain Questionnaire (MPQ) [9]. When the answer was “no”, they did not fill-out this questionnaire. To exclude other sources for pain symptoms, all existing comorbidities present at time of surgery were reported. The MPQ is a validated multidimensional pain questionnaire designed to measure the quality and intensity of chronic pain [10]. The main section of this questionnaire includes a list of 63 words, divided into three major classes: the sensory class, the affective class and the evaluative class. Pain intensity is measured quantitatively by the Number of Words Chosen (NWC), and qualitatively by the Pain Rating Index (PRI). The questionnaire includes a Visual Analogue Scale (VAS), to determine pain severity at the time of analysis. Lastly, the MPQ includes a section for localization, duration, and course of pain. The ‘onset of pain’ was one of the questions in the MPQ. This was subjectively filled in, and marks the way patients experienced the onset of the pain, i.e. like a sudden start in minutes/hours or a slow start in days/weeks.

Secondary outcomes were the prevalence of localized hypoesthesia, analysis of possible risk factors for CPSP and HRQoL. When patients answered “yes” to the localized hypoesthesia question they were included in the group with localized hypoesthesia and were asked to fill in the subsequent self-designed follow up questionnaire to determine its localization, duration, and course (Appendix 1). The ‘onset of numbness’ was one of the questions. This was subjectively filled in, and marks the way patients experienced the origination of the numbness feeling; i.e. fast (minutes/hours) or slow (days/weeks) onset.

All patients were asked to fill in the questionnaire regarding HRQoL: the RAND Short Form-36 Health Status Inventory (RAND SF-36) [11]. The RAND SF-36 is a validated questionnaire on HRQoL, separated into eight multi-item scales; physical functioning, role limitations due to physical health problems, role limitations due to emotional problems, general mental health, social functioning, energy/fatigue, bodily pain, and general health perceptions [11].

Perioperative patient data were collected from a prospectively maintained database and included: medical history, age at the time of surgery, BMI, ASA-score, medication and indication for surgery, side of adrenalectomy, duration of surgery, operating technique, conversion to open surgery and postoperative complications, according to the Clavien-Dindo classification. The presence of preoperative pain, preoperative anxiety disorders or depression was obtained from electronic patient documents and scored “yes” if it was reported.

### Statistical analysis

The data were assessed for normality using the Shapiro-Wilk Test. To compare normally distributed continuous variables Student *t*-test was used. Chi-square and ANOVA were used for categorical variables. Correlation between variables that were normally distributed was calculated with the Pearson correlation coefficient and with the Spearman rank correlation method for non-normally distributed variables.

To identify possible predictive factors for CPSP binary logistic regression was performed. The Hosmer-Lemeshow goodness of fit test was used to describe the performance of the regression model. Variables with a significance level of *p* < .05 in the univariate model were included in the multivariate model. Statistical significance was defined as a *p*-value <.05. All statistical analyses were performed using Statistical Package for the Social Sciences (SPSS IBM Statistics 24; Armonk, NY).

## Results

### Patient enrolment

**Fig. 1:**
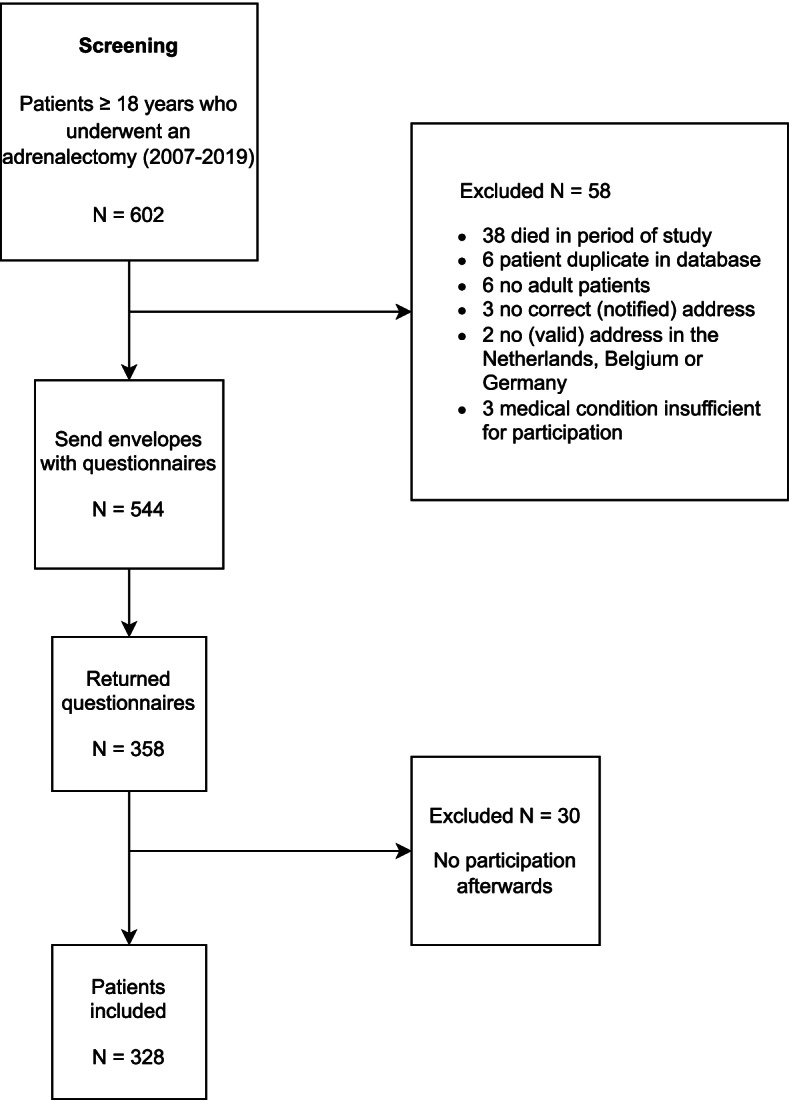
Patient enrolment

A total of 602 patients underwent MIA between February 2007 and September 2019. Five hundred forty-four patients were approached by phone between October 2019 and January 2020. Fifty-eight patients were lost to follow-up because of death or missing contact information. We received response from 358 (65.8%) patients, of which 328 patients signed informed consent. Of these patients, 172 underwent TLA and 156 PRA. Patient enrolment is depicted in Fig. 1.

### Patient characteristics

The mean age of patients at time of adrenalectomy was 54 years and 52% was male. The mean follow-up time was 4.6 years. The most common indications for surgery were primary aldosteronism (PA) (50%), pheochromocytoma (23%), and Cushing’s syndrome (13%). Forty-nine patients (15%) reported pain preoperatively, and 52 (16%) patients used pain medication preoperatively. In the group of patients with CPSP, 25% had reported pain preoperatively and 22% used analgesia before surgery. History or comorbidity of neurological diseases, which included for example cerebral vascular events, hernia nuclei pulposi, and peripheral neuropathy was not significantly different between patients with and without CPSP. Thirty-four patients had any postoperative complication, of which 15 (4.6%) patients had a Clavien-Dindo I complication, 11 (3.3%) patients had a Clavien-Dindo II, and 8 (2.4%) patients had a Clavien-Dindo III complication. Most Clavien-Dindo II complications were postoperative pulmonary infections or urinary tract infections (73%). The Clavien-Dindo III complications included three patients who underwent additional surgery (0.9%), two because of an incisional hernia and one because of an urinoma. Other Clavien-Dindo III complications included pulmonary embolism and postoperative abscess which required percutaneous drainage. Patient characteristics are presented in Table 1.

**Table 1:** Patient characteristics

	All patients *n* = 328	Patients with CPSP *n* = 49	Patients without CPSP *n* = 279
Age at time of surgery (y)	54.1 ± 12.3	50.6 ± 11.9	54.7 ± 12.2
Sex (male)	172 (52.4%)	20 (40.8%)	152 (54.5%)
BMI (kg m^− 2^)	27.8 ± 4.7	29.1 ± 5.9	27.5 ± 4.5
ASA-score, n (%)			
ASA 1	13 (4.0)	1 (2.0)	12 (0.7)
ASA 2	242 (73.8)	31 (63.3)	211 (75.6)
ASA 3	73 (22.3)	17 (34.7)	56 (20.1)
Diabetes Mellitus, n (%)	51 (15.5)	6 (12.2)	45 (16.1)
Preoperative pain, n (%)	49 (14.9)	12 (24.5)	37 (13.3)
Preoperative pain medication, n (%)			
None	276 (84,1)	38 (77.6)	238 (85.3)
Paracetamol	12 (3.7)	4 (8.2)	8 (2.9)
NSAID	8 (2.4)	3 (6.1)	5 (1.8)
Opioid	16 (4.9)	3 (6.1)	13 (4.7)
Other	16 (4.9)	1 (2)	15 (5.4)
Psychological: n (%)			
- Psychogenic gait disorder	2 (0.6)	1 (2)	1 (0.4)
- Depression	4 (1.2)	–	4 (1.4)
- Panic disorder	6 (1.8)	1 (2)	3 (1.1)
- Gilles de la Tourette	1 (0.3)	–	1 (0.4)
History of neurological disease, n (%)	89 (27.1)	13 (26.5)	76 (27.2)
History of abdominal surgery, n (%)	101 (30.8)	19 (38.8)	82 (29.4)
Indication of adrenalectomy, n (%)			
Primary aldosteronism	164 (50)	18 (36.7)	146 (52.3)
Pheochromocytoma	75 (23)	16 (32.7)	59 (21.1)
Cushing’s syndrome	43 (13)	8 (16.3)	35 (12.5)
Other	46 (14)	7 (14.3)	39 (14.0)
Side of adrenalectomy (left / right / both), n (%)	169 (51.5) / 139 (42.4) / 20 (6.1)	30 (61.2) / 16 (32.7) / 3 (6.1)	139 (50) / 123 (44.1) / 17 (6.1)
Type of procedure, n (%)			
Transperitoneal	172 (52.4)	34 (69.4)	138 (49.5)
Retroperitoneoscopic	156 (47.6)	15 (30.6)	141 (50.5)
Duration of surgery (min)	93.7 ± 44.3	99 ± 44.1	92.8 ± 44.4
Blood loss (mL)	47.7 ± 134.1	62 ± 164.7	45.2 ± 128,2
Tumor diameter (mm)	2.8 ± 2.3	3.5 ± 2.6	2.6 ± 2.2
Length of hospital stay (days)	3.8 ± 1.7	4.3 ± 2.1	3.7 ± 1.5
Conversion to transperitoneal /open procedure, n (%)	16 (4.9) / 2 (0.6)	1 (2) / 1 (2)	7 (2.5) / 5 (1.8)
Complications by Clavien-Dindo class, n (%)			
No complication	294 (89.6)	39 (79.6)	255 (91.4)
Clavien-Dindo I	15 (4.6)	3 (6.1)	12 (4.3)
Clavien-Dindo II	11 (3.3)	3 (6.1)	8 (2.9)
Clavien-Dindo III	8 (2.4)	4 (8.2)	4 (1.4)
Follow-up time since surgery, n (%)			
< 2 y	60 (18.3)	11 (22.4)	49 (17.6)
2–3 y	78 (23.8)	19 (38.8)	59 (21.1)
4–5 y	73 (22.3)	9 (18.4)	64 (22.9)
6–7 y	61 (18.6)	4 (8.2)	57 (20.4)
8–9 y	28 (8.5)	2 (4.1)	26 (9.3)
10–11 y	25 (7.6)	4 (8.2)	21 (7.5)
> 11 y	3 (0.9)	0	3 (1.1)

Categorical variables are presented as n (%); continuous variables are presented as mean ± SD

*ASA* American Society of Anesthesiologists*, BMI* Body mass index*, NSAID* Non-steroidal anti-inflammatory drug*, y* years

### Prevalence of chronic postsurgical pain

Forty-nine out of 328 patients (14.9%) reported the presence of CPSP, of which 34 received TLA (69.4%) and 15 PRA (30.6%). Onset of CPSP was acute in 18 patients (36.7%) and slow in 28 patients (57.1%). In the group of patients with CPSP, 32.7% reported hypoesthesia as well. In the group of patients with CPSP, most patients (44.9%) experienced continuous pain with fluctuating severity. In this group, the mean general pain intensity measured by VAS was 34 (± 25). The reported localizations were the ipsilateral flank (67.3%), the contralateral flank (16.3%) and other more diverse localizations such as groin, shoulders or arms (16.3%).

The mean Number of Words Chosen (NWC) was 7.9 of 20 words. Sensory descriptors were chosen more frequently than affective or evaluative terms. The most frequently selected words were nagging (*n* = 21; 42.9%) and stabbing (*n* = 20; 40.8%) in the sensory class; tiring (*n* = 25; 51%) in the affective class; and annoying (*n* = 26; 53.1%) in the evaluative class. The mean pain intensity measured by the total score of the PRI (PRI-Total; PRI-T) was 40.8 (SD 26.7; maximum score 63). Twenty patients (40.8%) were using analgesics for the reported pain, mostly acetaminophen or nonsteroidal anti-inflammatory drugs (34.7%); four patients reported the use of opioids (8.2%). Further characteristics are presented in Table 2.

**Table 2:** Characteristics CPSP and hypoesthesia

Questionnaire components	Patients with CPSP *n* = 49	Patients with hypoesthesia *n* = 52
Start of pain, n (%)		
Acute	18 (36.7)	28 (53.8)
Slow	28 (57.1)	18 (34.6)
Unknown	3 (6.1)	6 (11.5)
Both hypoesthesia and CPSP present, n (%)	16 (32.7%)	16 (30.8%)
Course of symptom experience, n (%)		
Attacks, with pain-free moments	14 (28.6)	–
Continuous, differing in severity	22 (44.9)	–
Continuous, stable severity	11 (22.4)	21 (40.4)
Unknown	2 (4.1)	31 (59.6)
VAS, general (0–100)	33.9 *±* 24.5	–
VAS, least pain (0–100)	13.3 *±* 18.0	–
VAS, worst pain (0–100)	56.7 *±* 23.0	–
Localization of pain, n (%)		
Ipsilateral flank	33 (67.3)	41 (78.8)
Contralateral flank	8 (16.3)	–
Other / unknown	8 (16.3)	11 (21.2)
Referred pain, n (%)	20 (40.8%)	–
Referred hypoesthesia, n (%)	–	7 (13.5%)
Tingling present, n (%)	–	23 (44.2%)
Pain intensity		
NWC-S (0 to 12 words)	3.9 ± 2.8	–
NWC-A (0 to 5 words)	1.8 *±* 1.7	–
NWC-E (0 to 3 words)	2.1 *±* 1.1	–
NWC-T (0 to 20 words)	7.9 *±* 5.0	–
PRI-S (0 to 36 words)	21.9 *±* 15.6	–
PRI-A (0 to 15 words)	9.1 *±* 8.7	–
PRI-E (0 to 12 words)	9.9 *±* 6.2	–
PRI-T (0 to 63 words)	40.8 *±* 26.7	–
Hypoesthesia feels annoying, n (%)		
No	–	33 (63.5)
Little	–	9 (17.3)
Fairly	–	6 (11.5)
Very	–	3 (5.8)
Unknown	–	1 (1.9)

Categorical variables are presented as n (%); continuous variables are presented as mean ± SD

*CPSP* Chronic postsurgical pain*, NWC-S* Number of Words Chosen of the sensory scale*, NWC-A* NWC of the affective scale*, NWC-E* NWC of the evaluative scale*, NWC-T* Total NWC*, PRI-S* Pain-Rating Index of the sensory scale*, PRI-A* PRI of the affective scale*, PRI-E* PRI of the evaluative scale*, PRI-T* Total PRI; VAS visual analogue scale

### Prevalence of hypoesthesia

Fifty-two patients (15.8%) reported hypoesthesia, of which 19 patients received TLA (36.5%) and 33 PRA (63.5%). Onset of hypoesthesia was acute in 28 patients (53.8%) and slow in 18 patients (34.6%). Twenty-one patients (40.4%) experienced a continuous feeling of hypoesthesia. The reported localizations were the ipsilateral flank (78.8%), leg or arms (11.5%) or unknown (9.6%). Further characteristics are presented in Table 2.

### Risk factors of CPSP

When looking at the univariate binary logistic regression, age, BMI, ASA-score, preexisting pain, surgical complications with Clavien-Dindo score III, TLA, and primary aldosteronism as indication for surgery were significant individual predictors of CPSP (Table 3). When performing multivariate binary logistic regression with these individual predictors, only young age remained a significant predictor for the development of CPSP. There was no collinearity between ASA-score and age. The Hosmer-Lemeshow goodness of fit test was not significant (*p* = .405).

**Table 3:** CPSP following MIA

Parameters	Univariate analysis OR (95% CI)	*p*-value	Multivariate analysis OR (95% CI)	*p*-value
Age at time of surgery	**0.973 (0.950–0.998)**	**.033**	**0.957 (0.930–0.983)**	**.002**
Gender (male)	0.576 (0.311–1.067)	.080	–	–
BMI (kg m^−2^)	**1.069 (1.006–1.137)**	**.031**	1.050 (0.984–1.121)	.139
ASA-score	**2.031 (1.099–3.751)**	**.024**	1.922 (0.955–3.871)	.067
Diabetes	0.726 (0.292–1.806)	.490		
Preoperative pain	**2.121 (1.015–4.434**	**.046**	1.607 (0.675–3.828)	.284
Preoperative neurological disease	0.965 (0.485–1.917)	.918		
History of abdominal surgery	1.522 (0.811–2.856)	.191		
Side of adrenalectomy (left)	1.659 (0.863–3.189)	.129		
Type of procedure (retroperitoneal)	**0.432 (0.225–0.828)**	**.011**	1.886 (0.941–3.779)	.074
Indication of adrenalectomy				
Primary aldosteronism	**0.529 (0.283–0.990)**	**.046**	0.637 (0.317–1.280)	.205
Pheochromocytoma	1.808 (0.932–3.507)	.080		
Cushing syndrome	1.360 (0.589–3.139)	.471		
Other	1.026 (0.430–2.445)	.954		
Duration of surgery (d)	1.003 (0.997–1.010)	.363	–	
Postoperative complications:				
Clavien-Dindo I	1.451 (0.394–5.342)	.576		
Clavien-Dindo II	2.209 (0.565–8.635)	.254		
Clavien-Dindo III	**6.111 (1.475–25.315)**	**.013**	3.091 (0.650–14.711)	.156

Significant *p*-values are in bold

*ASA* American Society of Anesthesiologists*, d* days*, MIA* Minimally invasive adrenalectomy*, OR* Odds ratio*, PA* Primary aldosteronism*, y* years*, 95% CI* 95% confidence interval

### Health related quality of life

The HRQoL of patients with CPSP after MIA was significantly lower in all subscales compared with patients without pain (Table 4). Major differences were seen in role limitations due to physical health problems between patients without CPSP (mean 76.1 ± SD 38.7) and patients with CPSP (mean 40.6 ± SD 43.3) (*p* < .001) and on the scale of bodily pain between patients without pain (mean 86.1 ± SD 21.9) and patients with CPSP (mean 59.1 ± SD 20.1) (*p* < .001). When analyzing pain intensity, a higher mean VAS-score was significantly correlated with a lower score on the following subscales of the RAND-SF36 questionnaire: role limitations due to physical health problems (R^2^ = 0.13, *p* = .012), role limitations due to emotional problems (R^2^ = 0.11, *p* = .024), general mental health (R^2^ = 0.18, *p* = .003) and bodily pain (R^2^ = 0.30, *p* = .000). In patients with CPSP the presence of hypoesthesia as well resulted in a significantly lower score of physical functioning (68.2 versus 42.2, *p* = .001). On the other seven domains there was no significant difference.

**Table 4:** Relationship between CPSP and HRQoL after adrenalectomy (RAND SF-36)

RAND SF-36 subscales (%)	Whole group *n* = 328	Patients with pain *n* = 49	Patients without pain *n* = 279	*p*-value
Physical functioning	78.3 ± 25.1	59.1 ± 26.2	81.5 ± 23.5	**<.0001**
Role limitations due to physical health problems	70.8 ± 41.3	40.6 ± 43.3	76.1 ± 38.7	**<.0001**
Role limitations due to emotional problems	83.1 ± 33.7	61.1 ± 43.1	86.9 ± 30.2	**<.0001**
Energy/fatigue	61.1 ± 20.8	44.9 ± 19.1	63.8 ± 19.9	**<.0001**
General mental health	76.4 ± 17.3	62.5 ± 20.6	78.9 ± 15.5	**<.0001**
Social functioning	83.6 ± 23.7	68.1 ± 27.4	86.2 ± 22.0	**<.0001**
Bodily pain	82.2 ± 23.6	59.1 ± 20.1	86.1 ± 21.9	**<.0001**
General health perceptions	58.5 ± 22.7	44.0 ± 20.2	61.0 ± 22.2	**<.0001**

Significant *p*-values are in bold. Variables are presented as mean ± SD

*HRQoL* Health-related quality of life

## Discussion

Although MIA was proven to be safe and effective for a heterogeneous group of patients with adrenal disorders, the prevalence of CPSP has not been reported widely. In this cohort study the prevalence of CPSP following MIA was 14.9%. The presence of CPSP was correlated with a significantly lower HRQoL.

*Acosta* et al. found a prevalence of 8% chronic back pain in twelve open and 6% in seventeen laparoscopic bilateral adrenalectomies for hypercortisolism [12]. *Walz* et al. observed an incidence of 8.5% of temporary hypoesthesia and/or relaxation of the abdominal wall after PRA [13]. A study by *Bruintjes* et al. showed a prevalence of CPSP of 5.7% following laparoscopic donor nephrectomy in relatively healthy live kidney donors. They also showed a significantly lower HRQoL in patients with CPSP on all subscales of the RAND-SF36, except role limitations due to emotional problems [14]. The prevalence of CPSP in our study was higher when compared to the study by *Bruintjes* et al. There are some possible explanations. First, there is a certain risk of recall bias, which might indicate that patient with pain symptoms are more willing to fill in the questionnaires compared to patients without pain, causing an overestimation of CPSP in this study. Second, we used the visual analogue scale and MPQ to report and classify postoperative pain. Differences in definition, classification systems used or methodology of analysis might impede comparisons among studies [15]. Third, patients with different comorbidities were analyzed in this study, compared to healthy donors in the study by *Bruintjes* et al. The prevalence of nerve injury-induced neuropathic pain was high in patients with persistent pain after thoracic and breast surgeries, 66 and 68%, respectively. In patients with CPSP after groin hernia repair, the prevalence of neuropathic pain was 31%, and after total hip or knee arthroplasty it was 6% [16]. So, the prevalence of nerve injury-induced neuropathic pain among CPSP cases differs in various types of surgery, probably depending on the likelihood of surgical iatrogenic nerve injury. Although, perioperative nerve injury seems to play an important role in the development of neuropathic pain, nociceptive and inflammatory processes can also be involved [16–18]. We found sixteen patients (33%) with a combination of CPSP and symptoms of hypoesthesia. This is in accordance with the study by *Johansen* et al., who reported a strong association between sensory abnormalities and persistent pain, increasing with higher pain intensities [19]. These findings may indicate that direct neuronal injury is a potential factor for developing CPSP, since nerve damage can result in central sensitization, which is linked to the development of CPSP [20]. Early prevention of central sensitization may provide a mechanism-based approach by blocking nociceptive input, for example by using regional anesthesia or through antihyperalgesic drugs, such as ketamine, subsequently reducing the chance to develop CPSP.

After multivariate regression analysis young age was a significant predictor of CPSP. This predictor has already been described for other surgical procedures than adrenalectomy, such as video-assisted thoracoscopy or thoracotomy [21], breast cancer surgery [22], and hysterectomy [23]. The etiology is not well-understood, but may be the result of a reduction in peripheral nerve functioning that occurs with increased age [24]. Recent publications describe a significant association between major postoperative complications and development of CPSP in much larger cohorts [25, 26]. The presence of a postoperative complications scored Clavien-Dindo III in our population was a significant predictor of CPSP in the univariate regression analysis, but not in the multivariate regression analysis. However, the result could be underpowered, since this group of patients was small.

When looking at pain severity, patients with a higher VAS-score had significantly lower scores on several domains of the RAND-SF36. This means that the presence of more severe pain results in a significantly lower HRQoL. CPSP can lead to functional limitations and psychological distress in patients. Therefore, identifying the risk factors and applying a preventive strategy may help to decrease the incidence of CPSP and the resulting lower HRQoL. Possible preventive strategies include modification of the surgical technique, adequate pain control throughout the perioperative period, and preoperative psychological intervention focusing on psychosocial and cognitive risk factors [27].

The main strength of this study is that we specifically investigated the prevalence of CPSP after MIA as a primary outcome, which was not done before. Furthermore, this study includes a large patient number from an expert centre, with a relatively high response rate compared with other small-scale phone or e-mail surveys [28]. This allowed us to perform multivariate logistic regression analyses to identify independent predictors of CPSP.

We acknowledge a few limitations in our study. First, patients with a variety of indications for surgery, disease-related symptoms and differences in comorbidity were compared. Preoperative patient data revealed that according to the group of patients with CPSP 25% had pre-existent pain symptoms, and 22% used analgesia before surgery. This could have an influence on preoperative and postoperative HRQoL between patients, and may influence their recovery after surgery, with or without the presence of CPSP. Second, because of the delay of inclusion (maximal 12 years from surgery to research contact), loss to follow-up may have influenced the results. We received response from 66% of patients, and 54% patients signed informed consent. Bias from lack of response might cause an incomplete analysis of the data. So, we cannot ensure that the remaining 46% had similar outcomes to those patients we assessed. Third, the questionnaire regarding CPSP was specific to only answer “yes” if the pain could be related back to their adrenalectomy, i.e. in time of onset and specific localization. However, it is still possible that patients with pre-existing pain reported “yes” as well. This could have resulted in an overestimation of the prevalence of CPSP. We tried to reduce this by searching and reporting comorbidities present at time of surgery which could lead to chronic pain symptoms. Finally, no structured preoperative HRQoL data were present in our population.

In conclusion, in this study we have shown a substantial prevalence of CPSP following MIA. The presence of CPSP was significantly correlated with a lower HRQoL. When present, CPSP should be identified in a timely manner, since adequate management by pharmacotherapy, appropriate pain interventions, and/or psychological management, can improve the pain and the physical and social functionality of patients. Furthermore, in the absence of evidence for the most effective treatment in established chronic pain, prevention should be the key strategy. Future trials should focus on etiology and prevention of CPSP after MIA.

## Supplementary Information

**Additional file 1.**

## Data Availability

The data that support the findings of this study are available on request from the corresponding author [EvH]. The datasets generated and/or analysed during the current study are not publicly available due to reasons of sensitivity, e.g. human data; containing information that could compromise research participant privacy.

## Abbreviations

ASA: American Society of Anesthesiologists
MIA: Minimally invasive adrenalectomy
TLA: Transperitoneal lateral approach
PRA: Posterior retroperitoneal approach
CPSP: Chronic postsurgical pain
HRQoL: Health-Related Quality of Life
BMI: Body mass index
MPQ: McGill Pain Questionnaire
RAND SF-36: RAND Short Form-36 Health Status Inventory
NWC: Number of Words Chosen
PRI: Pain Rating Index
VAS: Visual Analogue Scale
