# Supplementary material for: Chronic postsurgical pain after minimally invasive adrenalectomy: prevalence and impact on quality of life
Source: BMC Anesthesiol. 2022 May 19;22:153. doi: 10.1186/s12871-022-01696-4 (PMC9118616; doi:10.1186/s12871-022-01696-4)
Supplement: Supplementary file 1 — Additional file 1. [file 12871_2022_1696_MOESM1_ESM.docx]

**Supplemental material**

**APPENDIX 1: Supplemental Methods**

**We used the Dutch version “Questionnaire hypoesthesia”**

1. Do you currently have of have you had a numbness, dull feeling or tingling around the adrenal gland removal surgery area?

- Yes
- No

1. Please indicate (with a cross mark) on the drawing below where you have or have had a numb feeling?


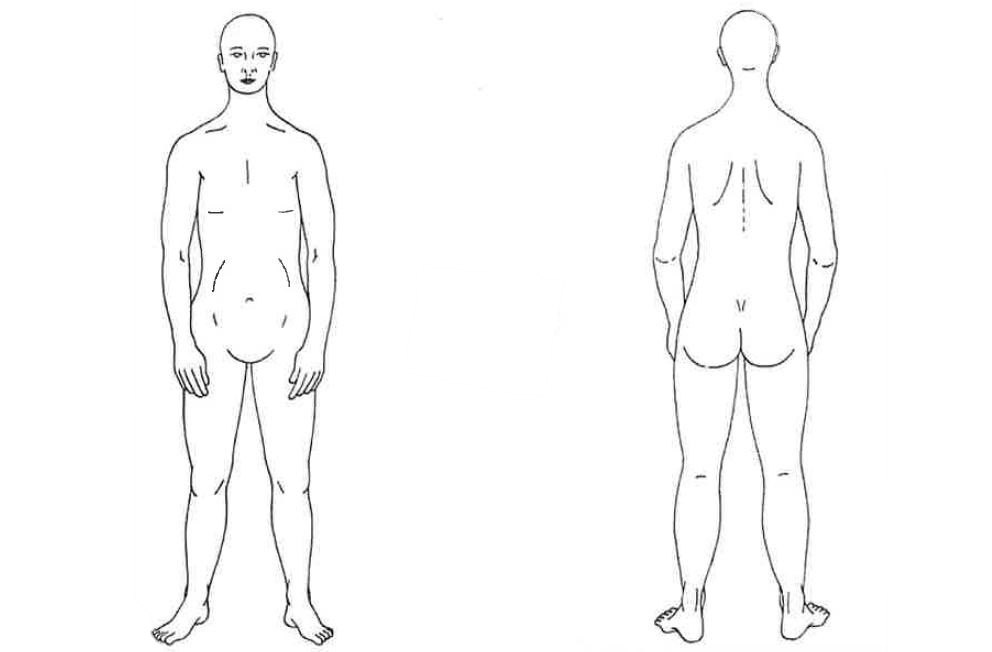


Below you will find a number of questions about the numbness that bothers you. If you feel a numb feeling in several places, complete the questions for the feeling that is the worst and most disturbing to you.

1. How long have you had this numbness feeling? …… year(s)

…… month(s)

…… week(s)

…… day(s)

1. How did the numbness feeling originate? Suddenly / Gradually
2. Is this numbness always in the same place(s)? Yes / No
3. Do you also feel tingling is this area? Yes / No
4. Is the numbness continuously present? Yes / No
5. Have you had a numb feeling in the past which Yes / No

has spontaneously improved?

1. If so, was this right after surgery? Yes / No
2. How long has the numbness lasted in total? ………..
3. Do you experience the numbness feeling as No / a little / quite / a lot

a nuisance in your daily activities?

1. Does it hinder you in carrying out your normal No / a little / quite / a lot

activities?

1. Does it hinder you from making normal movements? No / a little / quite / a lot
2. Did you feel lethargic because of this feeling No / a little / quite / a lot

yesterday?
